# Highly sensitive targeted methylome sequencing by post-bisulfite adaptor tagging

**DOI:** 10.1093/dnares/dsu034

**Published:** 2014-10-16

**Authors:** Fumihito Miura, Takashi Ito

**Affiliations:** 1 Department of Biophysics and Biochemistry, Graduate School of Science, University of Tokyo, Bunkyo-ku, Tokyo 113-0033, Japan; 2 Core Research for Evolutional Science and Technology (CREST), Japan Science and Technology Agency (JST), Higashi-ku, Fukuoka 812-8582, Japan; 3 Department of Biochemistry, Kyushu University Graduate School of Medical Sciences, Higashi-ku, Fukuoka 812-8582, Japan

**Keywords:** DNA methylation, target enrichment, massively parallel sequencing

## Abstract

The current gold standard method for methylome analysis is whole-genome bisulfite sequencing (WGBS), but its cost is substantial, especially for the purpose of multi-sample comparison of large methylomes. Shotgun bisulfite sequencing of target-enriched DNA, or targeted methylome sequencing (TMS), can be a flexible, cost-effective alternative to WGBS. However, the current TMS protocol requires a considerable amount of input DNA and hence is hardly applicable to samples of limited quantity. Here we report a method to overcome this limitation by using post-bisulfite adaptor tagging (PBAT), in which adaptor tagging is conducted after bisulfite treatment to circumvent bisulfite-induced loss of intact sequencing templates, thereby enabling TMS of a 100-fold smaller amount of input DNA with far fewer cycles of polymerase chain reaction than in the current protocol. We thus expect that the PBAT-mediated TMS will serve as an invaluable method in epigenomics.

## 1. Introduction

Methylation occurring at the position 5 of cytosine residues in DNA is an epigenetic modification critically involved in the regulation of eukaryotic genomes. Accordingly, the genome-wide distribution of 5-methylcytosine residues, or the methylome, has been attracting intense attention from a wide audience in a variety of research disciplines. The recent advent of next-generation sequencing has revolutionized the way of interrogating the methylome: it has realized whole-genome bisulfite sequencing (WGBS) or genome-wide methylation analysis at single-base resolution.^1,2^ The power of WGBS has been well demonstrated by many findings that would never have been achieved with other technologies. Although WGBS represents the gold standard for methylome analysis and is rapidly becoming the method of choice, its cost has remained substantial, thereby preventing it from being widely used for multi-sample comparison of large methylomes including those of mammals.

The most popular alternative to WGBS is reduced-representation bisulfite sequencing (RRBS), which efficiently enriches CpG-rich regions through restriction enzyme digestion to reduce the cost of sequencing, while maintaining deep coverage of a subset of CpG sites.^3^ Notably, RRBS can be applied to a minute amount of input DNA.^4^ However, it cannot be used to examine particular regions of interest unless they are adequately flanked by the restriction enzyme sites. In this context, the shotgun bisulfite sequencing of subgenomic regions enriched using solution hybridization capture technology, referred to hereafter as targeted methylome sequencing (TMS), is ideal, because it can in principle target any unique genomic region.^5–7^ However, all of the TMS protocols reported thus far require not only a considerable amount of input DNA (i.e. 3 µg or more), but also a large number of polymerase chain reaction (PCR) cycles (i.e. 10–20 cycles), making it difficult to apply TMS to samples of limited quantity.

We recently developed a highly efficient protocol for WGBS library construction termed post-bisulfite adaptor tagging (PBAT).^8^ Although it is well known that bisulfite treatment destroys DNA, all of the conventional ligation-based WGBS protocols as well as the tagmentation-based one^9^ treat adaptor-tagged library DNAs with bisulfite, inevitably leading to a considerable loss of intact sequencing template molecules. To circumvent this bisulfite-induced loss, we proposed the PBAT strategy in which adaptor tagging is performed after bisulfite treatment. This simple trick allowed us to prepare a PCR-free WGBS library from as little as 125 pg of input DNA.^8^ We routinely achieve a PCR-free, 30-fold coverage of mammalian methylomes from ∼30 ng of input DNA. Indeed, PBAT has been applied to mouse WGBS from 400 to 1,000 germinal vesicle-stage oocytes^10,11^ and a few thousand primordial germ cells,^12^ notably without any global PCR amplification. More recently, it has even been applied to single-cell genome-wide bisulfite sequencing with the aid of PCR.^13^

**Figure 1.**
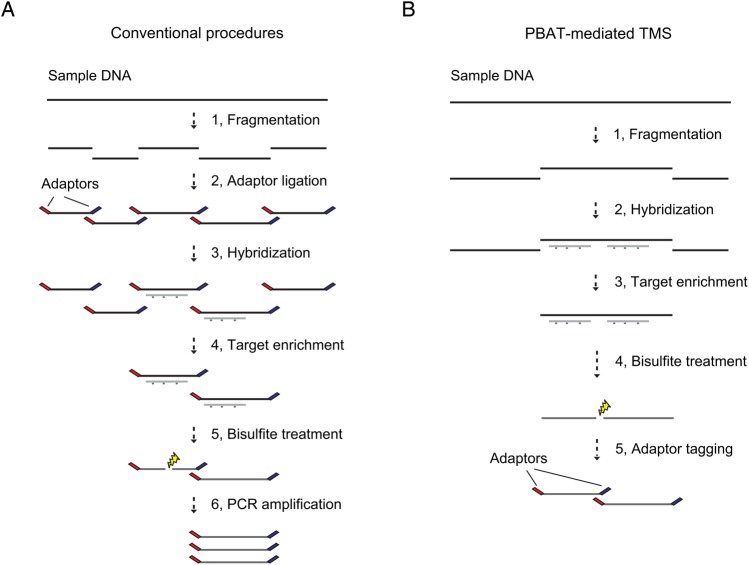
Two strategies for TMS. (A) Conventional procedures comprise adaptor tagging of fragmented genomic DNAs (Steps 1 and 2), target enrichment by hybridization (Steps 3 and 4) and bisulfite treatment of enriched library DNAs (Step 5) followed by PCR amplification (Step 6). The bisulfite treatment (Step 5) induces DNA breaks, inevitably leading to severe loss of intact sequencing template molecules. (B) PBAT-mediated procedure comprises target enrichment by hybridization (Steps 1–3), bisulfite treatment (Step 4) and adaptor tagging (Step 5), thereby circumventing the bisulfite-induced loss of intact sequencing template molecules.

As all of the current TMS protocols include bisulfite treatment of adaptor-tagged library DNA, we reasoned that PBAT can improve the efficiency of TMS to develop a low-input protocol (Fig. 1). We have indeed succeeded in the development of a highly efficient TMS method applicable to samples of limited quantity.

## 2. Materials and methods

### 2.1. Preparation of DNA

Both human and mouse genomic DNAs used in the model experiments were purchased from Promega. Genomic DNA from IMR90 primary human lung fibroblasts was a generous gift from Yae Kanai. The indicated amount of genomic DNA was dissolved in 130 µl of 10 mM Tris–HCl (pH 8.0) and sheared with Covaris S220 to the indicated size. We used AMPure XP to purify the fragmented DNA as follows. First, the shared DNA (130 µl) was mixed with 1.8× volume (234 µl) of the AMPure XP reagent and stood for 15 min at room temperature. Next, the beads were collected using a magnet stand, and the supernatant was removed. The pelleted beads were then rinsed with 70% ethanol and dried by standing at 37°C for 5 min. Finally, DNA was eluted from the beads to 20 µl of RNase-free water. The eluted DNA solution was dried in a vacuum concentrator and dissolved in 7 µl of RNase-free water.

### 2.2. Target enrichment

Enrichment of targets with liquid-phase hybridization capture was performed using the reagents in SureSelect Human or Mouse Methyl-Seq kit (Agilent). Genomic DNA (7 µl) fragmented and purified as above was supplemented with 3 µl of formamide (Wako, Biochemistry grade) and overlaid with 80 µl of mineral oil (Sigma). The DNA was completely denatured by incubating the tube at 99°C for 10 min, cooled down to 65°C and kept at 65°C for at least 5 min before adding the following reagents. Hybridization buffer was prepared by mixing 7.5, 0.3, 3.0 and 3.0 µl of Hyb#1, #2, #3 and #4, respectively. Capture probe mix was prepared by mixing 5.0, 0.5 and 1.0 µl of capture probe solution, RNase Inhibitor and RNase-free water, respectively. The hybridization buffer and the capture probe mix were individually overlaid with 80 µl of mineral oil and incubated at 65°C for 10 min. These two solutions were then combined and mixed thoroughly by pipetting. The combined solution was transferred to the tube containing the denatured input DNA kept at 65°C as above and mixed thoroughly with the DNA solution by pipetting. The tube was incubated at 65°C for at least 24 h to allow hybridization between the probes and the targets.

Fifty microlitres of well-suspended solution of DynaBeads MyOne Streptavidin T1 (Life Technologies) was taken into a 1.5-ml tube, and the beads were washed 2 times with 200 µl of Binding Buffer. To the pelleted beads, the hybridization reaction supplemented with 200 µl of Binding Buffer was added and mixed well. After incubation with rocking at room temperature for 30 min, the beads were collected using a magnetic stand and washed with 500 µl of Wash Buffer 1. The beads were then subjected to three rounds of washing, each composed of re-suspension in pre-warmed Buffer 2 followed by incubation at 65°C for 10 min. Following thorough removal of washing solutions from the tube, enriched DNA was eluted by incubating the beads in 20 µl of Elution solution at room temperature for 20 min. The eluate was immediately used for bisulfite treatment.

### 2.3. Bisulfite treatment

EZ DNA Methylation-Gold kit (Zymo Research) was used for the bisulfite treatment of target-enriched DNA, according to manufacturer's instruction. The enriched DNA solution (20 µl) was directly mixed with 130 µl of CT conversion reagent freshly prepared before use. The mixture was incubated at 64°C for 2.5 h. Note that the incubation step at 98°C for 10 min described in the instruction was omitted, because the target-enriched DNA was already denatured. Following the purification and desulfonation steps, bisulfite-treated DNA was eluted with 20 µl of M-Elution buffer.

### 2.4. PBAT library construction and Illumina sequencing

We used the bisulfite-treated DNA for library preparation according to the PBAT protocol^14^ (also available from http://crest-ihec.jp/english/epigenome/index.html), except for the primers used in the first- and second-strand synthesis. The primer used for the first-strand synthesis was 5′-ACA CTC TTT CCC TAC ACG ACG CTC TTC CGA TCT WWW WNN NN-3′ (W = A or T). The indexed primers used for the second-strand synthesis was 5′-CAA GCA GAA GAC GGC ATA CGA GAT XXX XXX GTA AAA CGA CGG CCA GCA GGA AAC AGC TAT GAC WWW WNN NN-3′, in which XXX XXX stands for the index sequence of each primer. We sequenced the constructed TMS libraries using Illumina HiSeq2500 as described previously.^14^

### 2.5. Data analysis

The obtained reads were mapped to human and mouse DNA using the hg19 and mm9 assemblies, respectively, analysed and visualized as described previously.^8^

## 3. Results

### 3.1. PBAT to target-enriched DNA

To test whether PBAT is applicable to TMS library construction, we applied it to target-enriched DNA prepared using the RNA probes provided in the Agilent SureSelect Mouse Methyl-Seq kit, which are designed to cover mouse genomic regions spanning 109 Mb in total. As PBAT employs random primer extension, it is ideal that the primer hybridizes to the 3′-flanking region of each target region in the input DNA and initiates the synthesis of a complementary DNA strand to fully span the target region. We therefore assumed the size of the input DNA to be critical and first examined its effects on the yield of the library. Starting from 3 µg of input DNA, as recommended by the manufacturer, we prepared DNA fragments with an average length of 180 bp, 400 bp or 2 kb (Supplementary Fig. S1A). From these DNA preparations, we enriched the targets by solution hybridization capture and successfully generated PCR-free single-read PBAT libraries, each of which was sufficient for one or more lanes in the HiSeq2500 system (Supplementary Fig. S1B). Note that the original Methyl-Seq protocol requires a total of 14 cycles of global PCR amplification when starting from the same amount of input DNA (i.e. 3 µg). As expected, the raw yield of library increased with the size of input DNA (Supplementary Fig. S1B). However, the normalized yield by the size of input DNA indicated that the 400-bp DNA fragments were most efficient. In addition, the library generated from the 2-kb DNA fragments contained a substantial number of off-target reads that were mapped near to but not onto the targets (Supplementary Fig. S1C). We therefore decided to use input DNA fragmented to a size of 400 bp for further development.

### 3.2. Novel primers for improved yield of indexed TMS libraries

It is highly likely that the users of TMS intend to sequence two or more indexed libraries in a single lane for efficient data collection. Thus, we next attempted to improve the efficiency of the PBAT paired-end/indexed read protocol, because it is 2-fold less sensitive than the single-read protocol.^14^ While the original PBAT protocol uses a random tetramer sequence (N_4_) attached to the 3′-end of primers containing Illumina adaptor sequences,^8^ we found that novel primers containing a semi-random tetramer composed solely of A or T bases immediately upstream of the random tetramer (W_4_N_4_; W = A or T) improved the yield of TMS library by ∼4-fold, with a marginal effect on the GC bias in target coverage (Supplementary Fig. S2). We also found that the addition of formamide to the hybridization capture reaction at a final concentration of 10% improved the reproducibility of the target enrichment step (data not shown). These modifications were critical to perform highly sensitive and robust construction of TMS libraries.

### 3.3. Characterization of low-input PBAT-mediated TMS libraries

**Figure 2.**
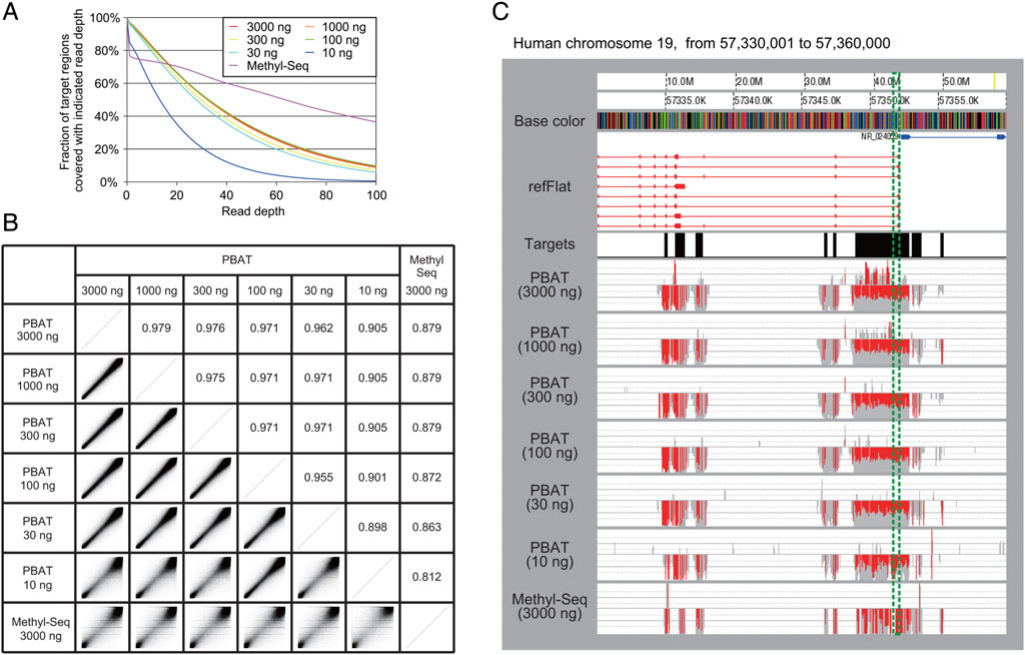
Performance of PBAT-mediated TMS. (A) Target coverage. The fraction of the targets covered by differing minimal depth of reads was shown for the six PBAT and one Methyl-Seq libraries generated from the indicated amount of input DNA. Note that the average read depth of the Methyl-Seq library was twice or more higher than those of the PBAT libraries (Table 1). (B) Consistency among TMS data. Methylation levels were compared among the six TMS libraries generated from 3,000 to 10 ng of human genomic DNA using the PBAT-mediated procedure as well as the one generated from 3,000 ng of input DNA using the original Methyl-Seq protocol (DRA002274-002280). The numbers and the images in the boxes above and below the diagonal indicated the coefficients of determination (*R*^2^) and the scatter plot of methylation levels, respectively, between all the possible combinations among the seven data sets. The moving averages of methylation levels (window size, 500 bp; step size, 250 bp) were calculated based on CpGs covered by 20 or more reads. (C) A snapshot of TMS data. Data around the imprinted control region (ICR) for *PEG3* were compared among the seven libraries generated with either PBAT or Methyl-Seq using the indicated amount of input DNA. Red bars and grey shadows indicated the methylation levels of individual CpG sites and the depth of reads, respectively. Note that most reads were mapped to the bottom strand, as the RNA probes used in the experiment were designed from the top strand. As expected, a region around the *PEG3* promoter showed ∼50% methylation level due to the imprinted monoallelic methylation. The green dashed box denoted the ICR of *PEG3* (chr.19: 57,351,728 to 57,352,173 in hg19 human reference genome sequence).

Using the protocol optimized as above, we prepared indexed TMS libraries from 3,000 to 10 ng of human and mouse DNA using the human and mouse RNA probe sets obtained from Agilent, respectively (Table 1). The yield of library DNA was nearly linearly correlated with the amount of input DNA (Table 1; Supplementary Fig. S3A). The smaller the amount of input DNA, the more evident the adaptor dimers were (Supplementary Fig. S3B). We performed the minimum cycles of PCR amplification to obtain sufficient DNA for a single HiSeq2500 lane (Table 1). We then used a half lane to sequence each of the human libraries and mapped the obtained reads to the reference human genome sequence. (Note that we combined the library DNA with the same amount of the PhiX control to compensate the extreme base bias of bisulfite-converted sequences.) The mapping rate was inversely correlated with the amount of input DNA within the range of 3,000 to 30 ng, but it showed a prominent decline when the DNA input was reduced to 10 ng (Table 1). In addition, all of the libraries except the one generated from 10 ng of DNA showed similar statistics regarding the coverage of the targets (Fig. 2A). The methylation levels of CpG sites covered by 20 or more reads were highly consistent among these five libraries (*R*^2^ > 0.95, when the window size and the step were 500 and 250 bp, respectively) (Fig. 2B). We inspected the methylation status of imprinted genes and found that the expected 50% methylation level was faithfully recapitulated, even in the library generated from 30 ng of DNA, but not in the library obtained from 10 ng of DNA (Fig. 2C). These results suggest that the PBAT-mediated TMS method is reliably applicable to as little as 30 ng of input DNA.

**Table 1.** Summary of library construction

Target species (Target size)	Method for library construction	DNA source	Amount of input DNA (ng)	Library yield before PCR amplification^a^ (amol)	Equivalent number of lanes^b^	Number of PCR cycles to have DNA enough for a single lane	Number of reads (% uniquely mapped reads)	Average depth of mapped reads obtained from a half lane of HiSeq2500 rapid mode		
								On target	Off target (near, ≤400 bp)	Off target (far, >400 bp)
Human (84 Mb)	Methyl-Seq	Promega human genomic DNA	3,000	1,340.0	1.00	11	110.3 M (91.1%)	98.2×	9.6×	0.2×
	PBAT		3,000	5,649.2	4.35	0	73.1 M (78.9%)	43.4×	9.3×	0.1×
			1,000	2,607.3	2.01	0	73.0 M (77.0%)	42.7×	8.9×	0.1×
			300	477.3	0.37	2	68.6 M (76.2%)	39.6×	8.2×	0.1×
			100	259.6	0.20	3	79.4 M (74.1%)	44.2×	9.2×	0.2×
			30	140.5	0.11	4	77.3 M (63.5%)	37.1×	7.9×	0.2×
			10	70.8	0.05	5	67.0 M (38.4%)	18.2×	4.1×	0.2×
		IMR90	300	220.0	0.17	4	70.8 M (85.6%)	46.3×	9.4×	0.1×
Mouse (109 Mb)	PBAT	Promega mouse genomic DNA	3,000	3,645.3	2.80	0	nd	nd	nd	nd
			1,000	2,353.3	1.81	0	nd	nd	nd	nd
			300	679.5	0.52	2	nd	nd	nd	nd
			100	285.2	0.22	3	nd	nd	nd	nd
			30	151.9	0.12	4	nd	nd	nd	nd
			10	71.2	0.05	5	nd	nd	nd	nd

M: million.

a Yield of the library amplified by 11 cycles of PCR was shown for Methyl-Seq.

b Based on an assumption that a single lane requires 1,300 amol of library DNA.

We also performed Methyl-Seq from the same human genomic DNA according to the protocol provided by the manufacturer, except that fewer PCR cycles were used (i.e. 11 cycles instead of 14 cycles), and compared the results with those of PBAT-mediated TMS. While the two data sets showed largely consistent methylation levels (*R*^2^ = 0.88) (Fig. 2B), they were distinct in terms of the GC content of the covered targets: Methyl-Seq and PBAT-mediated TMS preferentially covered AT-rich and GC-rich targets, respectively (Supplementary Fig. S4). This was presumably because the extensive global PCR step in Methyl-Seq failed to amplify GC-rich regions and because the random priming from the PBAT adaptor primers was rather compromised in AT-rich regions. We also note that the Methyl-Seq data recapitulated the methylation status of imprinted genes less precisely than the PBAT data (Fig. 2C).

**Figure 3.**
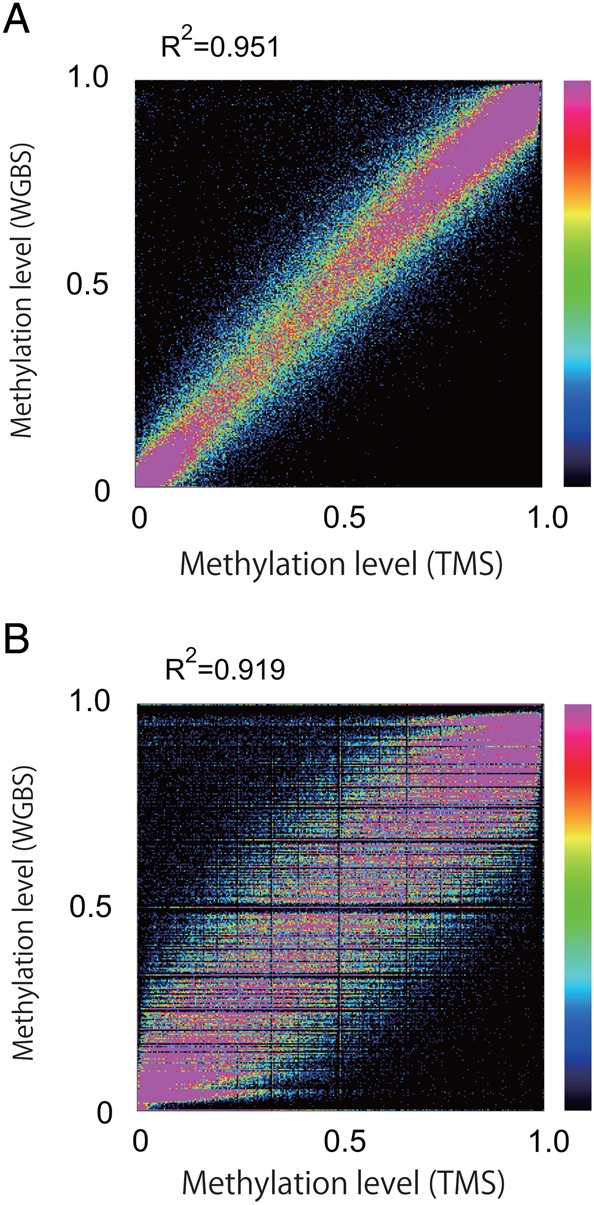
Consistency between TMS and WGBS data. Methylation levels were compared between the TMS data (DRA002281) (Table 1) and a publicly available WGBS data (DRA002248) on human IMR90 cells using the CpG sites covered by 20 or more reads. Methylation levels were plotted for moving windows (window size, 500 bp; stepping size, 250 bp) (A) and for individual CpG sites (B).

Having confirmed the high efficiency and consistency of PBAT-mediated TMS, we next compared it with WGBS. We performed TMS using 300 ng of genomic DNA from human IMR90 cells and compared the resulting data with PBAT-mediated WGBS data from the same cell line. The methylation levels of CpG sites covered by 20 or more reads showed an excellent correlation between the two data sets in either the window-based or the nucleotide-based analysis (Fig. 3). These results indicated that TMS can be a reliable alternative to WGBS.

## 4. Discussion

WGBS has become the gold standard method in methylomics for its unsurpassed resolution and coverage. However, it is too expensive to be used for analysing multiple samples. Efforts have thus been paid to develop cost-effective alternatives to WGBS, including various TMS methods based on padlock probes, array capture and solution hybridization capture.^7^ Among these approaches, the last one is highly flexible, but current protocols require a considerable amount of input DNA and are not applicable to samples of limited quantity.^5,6^ Furthermore, they include extensive global PCR amplification, which may increase the risk of inaccurate estimate of methylation levels, especially when the quantity of input DNA is limited. Therefore, a novel TMS protocol is desirable that requires a much smaller amount of input DNA with far fewer cycles of global PCR amplification than current ones. As the inefficiency of current protocols is likely attributable to bisulfite-induced degradation of target-enriched library DNA (Fig. 1A), we reasoned that the PBAT strategy can circumvent the adverse effect of bisulfite treatment to improve the efficiency of TMS (Fig. 1B).

We tested the possibility using the biotinylated RNA probes in the Agilent Methyl-Seq kit designed to cover CpG islands with their shores and shelves, enhancers, promoters, differentially methylated regions and other regulatory elements. Our results demonstrated that PBAT enables TMS from a 100-fold smaller amount of input DNA (i.e. 30 versus 3,000 ng) with considerably fewer cycles of PCR amplification (i.e. 4 versus 11–14 cycles) than the original protocol provided by the manufacturer (Table 1; Fig. 2). It also enables PCR-free TMS, provided that 1 µg of input DNA is available. Furthermore, the coverage statistics and the methylation levels of imprinted genes indicated that the PBAT-mediated protocol has superior coverage and accuracy than the original one (Fig. 2). We also confirmed high consistency between PBAT-mediated TMS and WGBS (Fig. 3), proving that the former can serve as a reliable surrogate for the latter. Although further improvements are necessary to generate high-quality TMS libraries from <30 ng of input DNA and to achieve more even coverage of the targets regardless of their GC contents, the PBAT-mediated protocol significantly outperforms the original Methyl-Seq protocol. It will be also useful for base-resolution analysis of 5-hydroxymethylcytosine when applied to genomic DNA treated with adequate oxidants or enzymes.^15,16^

We also evaluated the cost performance of PBAT-mediated TMS in comparison with those of PBAT-mediated WGBS and RRBS. The TMS is ∼10 times less expensive than the WGBS, when the former and the latter use the high output mode of HiSeq to achieve 40-fold coverage of the target regions and the whole genome, respectively. Note that, the deeper the coverage is, the more cost effective the TMS is. While the target regions in the TMS cover only ∼2.8% of the human genome, they include approximately one-seventh of the total CpG sites. On the other hand, the TMS costs 4–5 times more but covers only ∼1.5 times more CpG sites than RRBS does. Nevertheless, the TMS likely remains highly competitive to RRBS in many instances, since the former covers a much broader range of genomic elements than the latter.

Taken together, PBAT significantly enhances the utility of TMS to enable various novel applications, especially those analysing a large number of precious samples. The PBAT-mediated TMS will thus serve as an invaluable tool for epigenomics.

## 5. Availability

The TMS data sets from this study have been submitted to the DDBJ Sequence Read Archive under the accession numbers DRA002274-002281.

## Supplementary data

Supplementary data are available at www.dnaresearch.oxfordjournals.org.

## Funding

This work was supported by Research Program of Innovative Cell Biology by Innovative Technology (Cell Innovation) (to T.I.), Platform for Drug Discovery, Informatics and Structural Life Science (to T.I.) and Grant-in-Aid for Scientific Research on Innovative Areas (25129702) (to F.M.) from the Ministry of Education, Culture, Sports, Science and Technology (MEXT) of Japan. Funding to pay the Open Access publication charges for this article was provided by MEXT.

## Supplementary Material

Supplementary Data

## References

[1] Cokus S.J., Feng S., Zhang X. (2008). Shotgun bisulphite sequencing of the *Arabidopsis* genome reveals DNA methylation patterning. Nature.

[2] Lister R., O'Malley R.C., Tonti-Filippini J. (2008). Highly integrated single-base resolution maps of the epigenome in *Arabidopsis*. Cell.

[3] Meissner A., Gnirke A., Bell G.W., Ramsahoye B., Lander E.S., Jaenisch R. (2005). Reduced representation bisulfite sequencing for comparative high-resolution DNA methylation analysis. Nucleic Acids Res..

[4] Gu H., Smith Z.D., Bock C., Boyle P., Gnirke A., Meissner A. (2011). Preparation of reduced representation bisulfite sequencing libraries for genome-scale DNA methylation profiling. Nat. Protoc..

[5] Lee E.J., Pei L., Srivastava G. (2011). Targeted bisulfite sequencing by solution hybrid selection and massively parallel sequencing. Nucleic Acids Res..

[6] Wang J., Jiang H., Ji G. (2011). High resolution profiling of human exon methylation by liquid hybridization capture-based bisulfite sequencing. BMC Genomics.

[7] Lee E.J., Luo J., Wilson J.M., Shi H. (2013). Analyzing the cancer methylome through targeted bisulfite sequencing. Cancer Lett..

[8] Miura F., Enomoto Y., Dairiki R., Ito T. (2012). Amplification-free whole-genome bisulfite sequencing by post-bisulfite adaptor tagging. Nucleic Acids Res..

[9] Adey A., Shendure J. (2012). Ultra-low-input, tagmentation-based whole-genome bisulfite sequencing. Genome Res..

[10] Kobayashi H., Sakurai T., Imai M. (2012). Contribution of intragenic DNA methylation in mouse gametic DNA methylomes to establish oocyte-specific heritable marks. PLoS Genet..

[11] Shirane K., Toh H., Kobayashi H. (2013). Mouse oocyte methylomes at base resolution reveal genome-wide accumulation of non-CpG methylation and role of DNA methyltransferases. PLoS Genet..

[12] Kobayashi H., Sakurai T., Miura F. (2013). High resolution DNA methylome analysis of primordial germ cells identifies gender-specific reprogramming in mice. Genome Res..

[13] Smallwood S.A., Lee H.J., Angermueller C. (2014). Single-cell genome-wide bisulfite sequencing for assessing epigenetic heterogeneity. Nat. Methods.

[14] Miura F., Ito T. (2014). PBAT: post-bisulfite adaptor tagging for highly sensitive whole-genome bisulfite sequencing. Methods Mol. Biol..

[15] Booth M.J., Branco M.R., Ficz G. (2012). Quantitative sequencing of 5-methylcytosine and 5-hydroxymethylcytosine at single-base resolution. Science.

[16] Yu M., Hon G.C., Szulwach K.E. (2012). Base-resolution analysis of 5-hydroxymethylcytosine in the mammalian genome. Cell.

