# Supplementary material for: Highly sensitive targeted methylome sequencing by post-bisulfite adaptor tagging
Source: DNA Res. 2014 Oct 16;22(1):13–8. doi: 10.1093/dnares/dsu034 (PMC4379973; doi:10.1093/dnares/dsu034)
Supplement: Supplementary Data [file supp_22_1_13__index.html]

Highly sensitive targeted methylome sequencing by post-bisulfite adaptor tagging — Supplementary Data 

# Highly sensitive targeted methylome sequencing by post-bisulfite adaptor tagging

## Supplementary Data

Supplementary Data

**Files in this Data Supplement:**

- Supplementary Figures - pdf file
