# Supplementary material for: Highly sensitive targeted methylome sequencing by post-bisulfite adaptor tagging
Source: DNA Res. 2014 Oct 16;22(1):13–8. doi: 10.1093/dnares/dsu034 (PMC4379973; doi:10.1093/dnares/dsu034)
Supplement: Supplementary Data [file supp_dsu034_dsu034supp_figures.pdf]

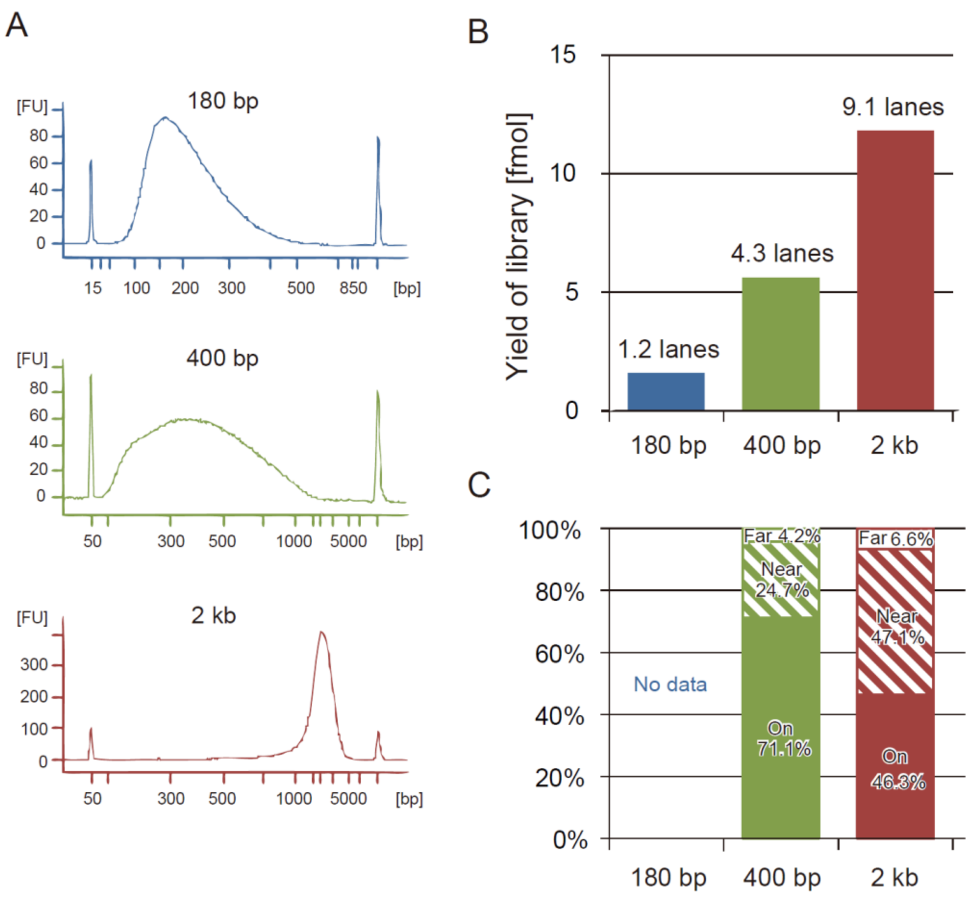

Supplementary Figure S1. Effect of input DNA size on the yield and quality of TMS library.

(A) Size distribution of the mouse genomic DNAs used for the experiment. Each DNA sample was fragmented with Covaris S220 under the indicated size setting and separated on Agilent Bioanalyzer 2000.

(B) Yields of the PBAT-mediated TMS libraries generated from 3 µg of fragmented DNAs. The amount of library DNA was quantitated with qPCR and indicated as the equivalent number of HiSeq2500 lanes. If the size of input DNA affects neither the efficiency of hybridization capture nor PBAT, the yield should be linearly correlated with the size of input DNA fragments. In other words, the yield should be identical when normalized by the size of input DNA. However, the normalized yield of the library generated from the 400 bp DNA fragments (*i.e.*, 10.75 lanes/kb) was significantly higher than those of the other two libraries (*i.e.*, 6.67 lanes/kb for 180 bp, and 4.55 lanes/kb): the 400 bp fragments outperformed the others as the input DNA.

(C) Mapping results of two TMS libraries. On, mapped onto the target regions; Near, mapped to the 2 kb-flanking regions upstream and downstream of the targets; Far, mapped to the regions >2 kb distant from the targets.

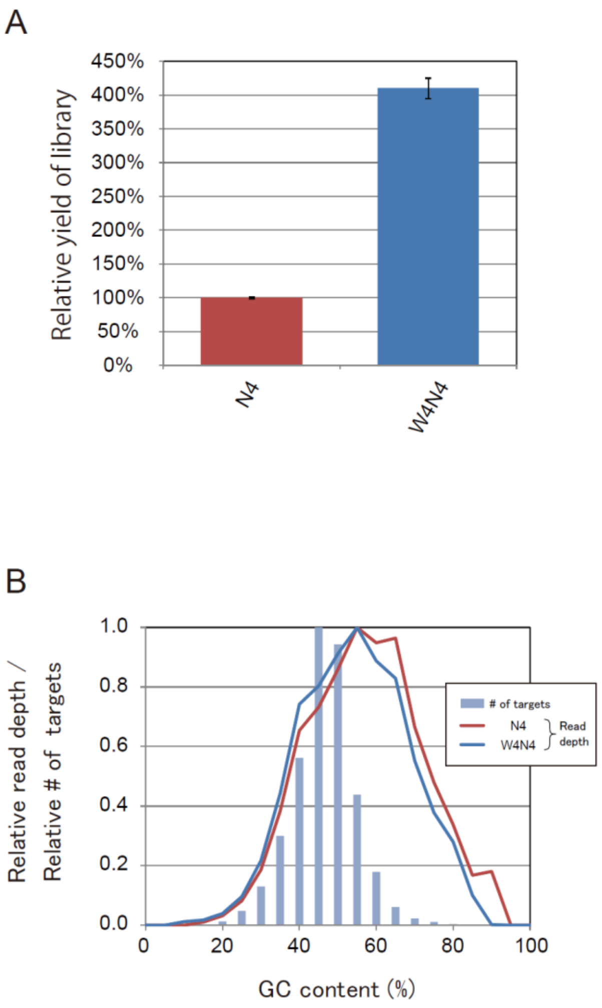

Supplementary Figure S2. Effect of primers on the yield of TMS library.

(A) Yields of PBAT-mediated TMS libraries generated using the conventional primer (N_4) and a novel primer (W_4N_4).

(B) Effect of GC-content on the coverage. Normalized read depth was plotted against the GC-content of the target regions. Red and blue lines indicated the data obtained using the N_4 and W_4N_4 primers, respectively. The vertical bar indicated the fraction of target regions in each GC-content window.

A

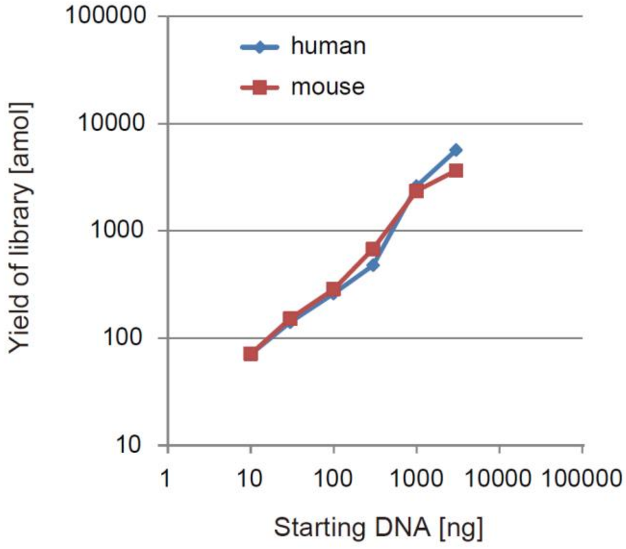

B

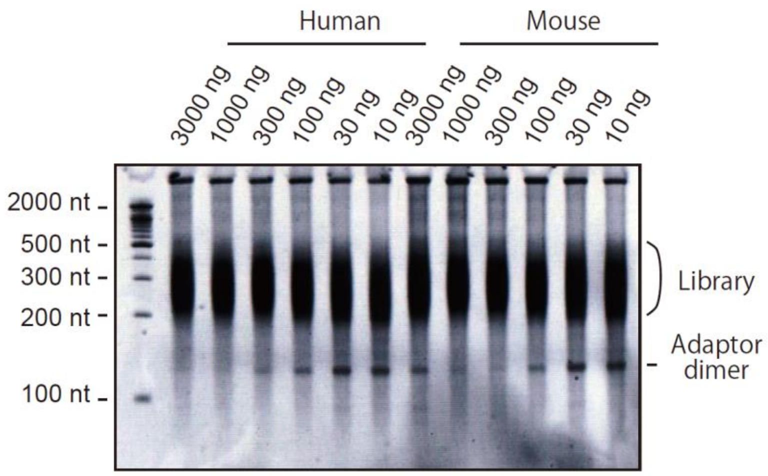

Supplementary Figure S3. Effect of input DNA amounts on TMS library.

(A) Relationship between the amounts of input DNA and library DNA. Data shown in Table 1 were plotted for human and mouse PBAT-mediated TMS libraries.

(B) Size distribution of library DNA. Each library was quantitated with qPCR, and the amplified products were separated with denaturing polyacrylamide gel electrophoresis and visualized with SYBR Gold staining.

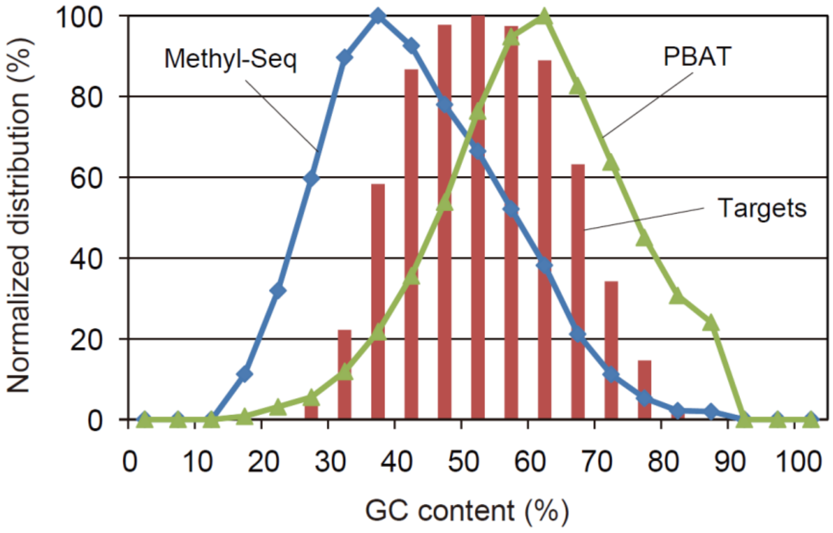

Supplementary Figure S4. GC-bias in target coverage.

Normalized read depth was plotted against the GC-content of the target regions for the Methyl-Seq and PBAT libraries generated from 3,000 ng of human DNA (Table 1).
